# The social relevance of occupational medical examinations in Brazil

**DOI:** 10.47626/1679-4435-2026-1611

**Published:** 2026-09-01

**Authors:** Eduardo Myung, José Domingos-Neto, Fernando Akio Mariya, Alexander Buarque, Ricardo Lupianhes Pacheco, Marcia Bandini

**Affiliations:** 1 Universidade de São Paulo (USP), São Paulo, SP, Brazil; 2 Hospital Alemão Oswaldo Cruz (HAOC), São Paulo, SP, Brazil; 3 Centro de Estudos Augusto Leopoldo Ayrosa Galvão (CEALAG), São Paulo, SP, Brazil; 4 Associação Brasileira de Empresas de Segurança e Saúde no Trabalho (ABRESST), São Paulo, SP, Brazil; 5 Universidade Estadual de Campinas (UNICAMP), Faculdade de Ciências Médicas, Campinas, SP, Brazil

**Keywords:** occupational health program, occupational health policy, occupational health, public health

## Abstract

Brazilian Bill No. 1,083/2021 highlights the need to reflect on the foundations, values, and objectives of occupational medical examinations. This essay, addressed to occupational physicians as well as other members and leaders of Brazilian society, discusses the importance and fundamental objectives of occupational medical examinations as an epidemiological and preventive tool aimed at promoting, monitoring, and conducting surveillance of workers’ overall health, with the potential to benefit public health and Brazilian society. The objectives and potential of occupational medical examinations extend beyond their medicolegal functions related to assessing fitness for work or establishing a causal relationship between disease and work. Occupational medical examinations are not an end in themselves but rather an entry point to multiple care pathways aimed at the prevention, monitoring, and comprehensive health care of workers. These actions encompass both asymptomatic workers exposed to risk factors for illness and those who already exhibit early signs of adverse health effects, while also including preventive ergonomic interventions, investigations of occupational accidents and work-related diseases, among others. The planning of these care pathways, priority setting, resource allocation, and evaluation of their effectiveness depend on epidemiological data obtained through occupational medical examinations. In this context, technical position statements issued by institutions representing occupational health can contribute to building consensus and advancing best practices related to occupational medical examinations. The authors advocate the rejection of Brazilian Bill No. 1,083/2021, as they consider its approval a setback in the historical evolution of occupational medical examinations.

## INTRODUCTION

Brazilian Bill No. 1,083/2021 calls into question the quality and necessity of occupational medical examinations by proposing that they be mandatory only for certain higher-risk occupational activities and for specific groups of workers, such as older adults, pregnant women, and individuals with disabilities, based on the premise that these examinations impose unnecessary costs on employers. However, the quality and structure of the Brazilian Occupational Health Medical Control Program (PCMSO, in Portuguese) and occupational medical examinations vary substantially across companies, resulting in different levels of understanding and engagement among workers, employers, and physicians regarding their objectives and purposes. Thus, the Bill underscores the need to reflect on the foundations, values, and objectives of occupational medical examinations, as well as on the validity of the criticisms and proposals it advances.

This essay, addressed to occupational physicians as well as other members and leaders of Brazilian society, proposes such a reflection through the presentation and discussion of excerpts from the Bill No. 1,083/2021 and from the opinion issued by the Brazilian Health Committee (CSAUDE, in Portuguese) regarding this legislative proposal. In addition, it provides a brief historical overview of the legislation governing occupational medical examinations in Brazil, analyzes the practical application of the current version of Regulatory Standard No. 7 (NR-7), discusses the social and strategic relevance of occupational medical examinations in the practice of Occupational Medicine, and offers recommendations for improving the design of the PCMSO and the conduct of occupational medical examinations.

### BILL NO. 1,083/2021 QUESTIONS THE NECESSITY AND QUALITY OF OCCUPATIONAL MEDICAL EXAMINATIONS

Bill No. 1,083/2021, introduced by Federal Deputy Kim Kataguiri on March 26, 2021, proposes to “amend the Consolidation of Labor Laws to eliminate the mandatory requirement for periodic, pre-employment, and pre-termination medical examinations,” such that “medical examinations will only be performed when essential to workers’ health, such as in cases involving older workers, pregnant women, individuals with disabilities, or when the work to be performed is hazardous, unhealthy, or arduous. For activities that do not have these characteristics, medical examinations will no longer be required.” In its justification, the Bill No. 1,083/2021 emphasizes the need to “reduce bureaucracy in labor relations, making hiring less expensive and simpler,” while also alleging the existence of “an occupational medicine industry that is sustained by conducting examinations that are, more often than not, superficial and unnecessary.”

Bill No. 1,083/2021 adopts an approach to the PCMSO and occupational medical examinations that is disconnected from the principles of public health, epidemiology, prevention, health surveillance, and comprehensive workers’ health management by excluding from occupational medical examinations a substantial proportion of the economically active population exposed to individual and occupational risk factors. Furthermore, it is based on a conception of occupational risk factors that is not supported by the epidemiological indicators of the Brazilian National Institute of Social Security (INSS, in Portuguese) or by the scientific literature, both of which demonstrate the occurrence of work-related health conditions among young workers and those engaged in administrative occupations, such as in the banking sector [^1^,^2^]. Likewise, the proposal is inconsistent with the Occupational Health Services Recommendation, 1959 (No. 112), issued by the International Labour Organization. Selected excerpts from the CSAUDE opinion on Bill No. 1,083/2021 are presented below, accompanied by commentary.

### CSAUDE ISSUES AN OPINION SUPPORTING THE MAINTENANCE OF MANDATORY OCCUPATIONAL MEDICAL EXAMINATIONS

In response to Bill No. 1,083/2021, Federal Deputy Ruy Carneiro, rapporteur of the CSAUDE, issued an opinion supporting the continued mandatory requirement for occupational medical examinations. Selected excerpts from that opinion are presented below:

*“Medical examinations performed in the occupational setting should not be viewed simply as bureaucratic and dispensable procedures. They are of great importance, safeguarding the rights not only of workers but also of employers.*

*Through the pre-employment medical examination, employers can determine whether a job applicant has a preexisting disease prior to the employment relationship, thereby protecting themselves against allegations that a disease was caused by work.*

*In this regard, an employer who performs a “superficial” pre-employment medical examination – as described by the distinguished Federal Deputy who authored the proposal, is effectively producing evidence against themselves, as they are certifying that the worker has no preexisting diseases and that any disease identified during or upon termination of the employment relationship has a temporal relationship with the work performed.*

*For workers, the pre-employment medical examination informs employers of preexisting diseases prior to the employment relationship, thereby protecting workers from being assigned to activities that could worsen their health condition.*

*Furthermore, the protection of workers is indispensable, non-negotiable, and enshrined in the 1988 Federal Constitution, with the pre-employment medical examination serving as evidentiary support in cases involving diseases that develop as a result of work.*

*Even when there is generally no causal relationship between the onset of a disease in a worker and the work performed, for example, in the case of a chronic disease diagnosed during the employment relationship, it is undeniable that the work performed may aggravate the worker’s condition. For this reason, periodic medical examinations are highly relevant and encourage investments in the quality of working conditions and workers’ quality of life.*

*Finally, the pre-termination medical examination is also of paramount importance for employers, as it demonstrates that, at the time the employment relationship ends, the worker was in good health and that any disease diagnosed thereafter is, in principle, unrelated to the work performed.”*

The CSAUDE opinion highlights that the quality of the PCMSO structure and occupational medical examinations is also in the employer’s interest. Employers are responsible for selecting the teams of the Brazilian Specialized Service in Safety Engineering and Occupational Medicine (SESMT, in Portuguese) and for influencing the budget, scope, and acceptance of the PCMSO within the company. Consequently, when employers, whether due to a lack of knowledge or interest, fail to invest in or require the SESMT to improve the structure of the PCMSO and the conduct of occupational medical examinations, they forfeit the opportunity to implement a genuinely preventive approach to workers’ health management guided by epidemiological evidence. The following section provides a historical overview of the legislation governing occupational medical examinations in Brazil.

### A BRIEF HISTORICAL OVERVIEW OF THE LEGISLATION GOVERNING OCCUPATIONAL MEDICAL EXAMINATIONS IN BRAZIL

Occupational medical examinations were formally incorporated into Brazilian legislation with the enactment of Law No. 6,514 of December 22, 1977, which amended Articles 168 and 200 of Decree-Law No. 5,452 of May 1, 1943 (Brazilian Consolidation of Labor Laws [CLT, in Portuguese]). The revised wording of Article 168 made employer-funded medical examinations mandatory for employees, while Article 200 granted the then Ministry of Labor the authority to establish supplementary provisions to the CLT.

However, as early as 1967, Decree-Law No. 229 of February 28 had already amended Article 167 of the CLT by requiring routine miniature chest radiography (abreugraphy) for all workers. Developed by the Brazilian physician Manoel Dias de Abreu, this radiographic technique was intended to provide rapid, low-cost screening for tuberculosis and represents a historical example of the use of occupational medical examinations to advance public health [^3^].

Subsequently, Ordinance No. 3,214, issued by the Ministry of Labor on June 8, 1978, established the NRs. In its original version, NR-7, entitled Medical Examinations, established the basic parameters for conducting occupational medical examinations.

NR-7 was subsequently revised by Ordinance No. 12 of the Secretariat of Occupational Safety and Medicine, issued on June 6, 1983, which defined the objectives of occupational medical examinations as obtaining clinical and occupational histories, assessing physical and mental fitness for work, requesting complementary tests for the surveillance and monitoring of workers’ health, and referring workers to the National Institute of Social Providence in suspected or confirmed cases of occupational or work-related disease.

Later, Ordinance No. 3,720 of the Ministry of Labor and Social Security, issued on October 31, 1990, removed abreugraphy from the mandatory examinations required under NR-7, in accordance with the guidelines then in effect from the Ministry of Health and the World Health Organization [^3^].

The first major structural revision of NR-7 was introduced through Ordinance No. 24 of the Secretariat of Occupational Safety and Health of the Ministry of Labor, issued on December 29, 1994, which established the mandatory implementation of the PCMSO with the objective of promoting workers’ health and preventing disease. Following this revision, occupational medical examinations became part of a planned and structured program based on workplace hazards identified through the other instruments established by the NRs.

This historical evolution of the legislation and the NRs highlights the progressive development of occupational health frameworks and professional consensus in Brazil. The following section discusses the practical application of the current version of NR-7 to evaluate the validity of the arguments advanced by Bill No. 1,083/2021.

### THE PRACTICAL APPLICATION OF THE CURRENT VERSION OF NR-7

The current version of NR-7 was established by MTP Ordinance No. 567 of March 10, 2022. Section 7.3.1 establishes that the PCMSO is an integral component of a broader set of organizational initiatives aimed at protecting workers’ health, operating in coordination with the recommendations and measures established by the other NRs.

Section 7.3.2 explicitly defines the preventive and epidemiological nature of the PCMSO and occupational medical examinations.

The following are illustrative examples of programs, protocols, and initiatives that some companies implement as part of the PCMSO, consistent with Sections 7.3.2.a through 7.3.2.l of NR-7, demonstrating the breadth and practical relevance of occupational medical examinations.

Section 7.3.2.a – Early screening and detection of work-related health conditions. In practice, some PCMSOs incorporate standardized screening instruments into periodic medical examinations to identify chronic pain, depression, and anxiety, together with systematic documentation of abnormalities identified during orthopedic examinations and complaints related to work organization. These data facilitate the early implementation of treatment, multidisciplinary follow-up, workplace accommodations, and investigation of potential associations between disease and work [^4^].

Section 7.3.2.b – Detection of excessive exposure to harmful occupational agents. Some PCMSOs include questionnaires designed to assess working conditions and work organization, enabling the identification of problems related to workplace furniture, excessive workload, occupational violence, and workplace bullying. During occupational medical examinations, occupational physicians may advise workers regarding the appropriate institutional channels for investigating and mitigating these situations, including reporting mechanisms, conflict mediation, ergonomic interventions, and requests for improvements to the work environment [^5^].

Section 7.3.2.c – Determination of each employee’s fitness for work. Occupational medical examinations may identify workers with medical conditions associated with an increased risk of occupational accidents or functional impairment. Individuals with obstructive sleep apnea or psychotic disorders, for example, may present an elevated occupational risk and require specialized follow-up through Occupational Medicine. Moreover, some workers with disabilities are identified for the first time during occupational medical examinations [^6^,^7^].

Section 7.3.2.d – Supporting epidemiological and statistical analyses of health conditions and their relationship with occupational risks. When properly recorded in electronic information systems, data obtained from occupational medical examinations enable the development of epidemiological indicators, the establishment of comparison groups, and studies investigating risk factors, thereby supporting preventive measures and assessments of causal relationships. For example, workers who smoke may have a higher incidence of lateral epicondylitis [^8^], a condition that is also influenced by the biomechanical demands of work [^9^]. These findings support smoking cessation programs, strengthen causal assessments, and improve technical communication between occupational physicians and employers.

Section 7.3.2.g – Supporting the reporting of work-related health conditions. During any occupational medical examination, suspected or confirmed cases of work-related diseases, Workers’ Compensation Accident Reports issued by other institutions, or occupational benefits granted by the INSS may be identified. Appropriate management of these cases requires protocols integrated into the PCMSO, adapted to local circumstances, and aligned with Resolution No. 2,323/2022 of the Brazilian Federal Council of Medicine (CFM, in Portuguese). In addition, Section 17.3.2 of NR-17 requires that an ergonomic work analysis (AET) be conducted in cases involving work-related diseases.

Sections 7.3.2.h through 7.3.2.k – Integrated coordination among workers, employers, attending physicians, and the Social Security system. Some companies have implemented structured return-to-work programs to monitor workers following the termination of Social Security benefits [^10^]. Return-to-work examinations enable a biopsychosocial assessment of work capacity by identifying prognostic factors, barriers to self-care, and the need for individual and occupational interventions. For example, a worker with an anxiety disorder may require support in interpersonal conflict mediation or organizational accommodations to facilitate a successful return to work.

Section 7.3.2.l – Monitoring employees’ active immunization. A recent example of the strategic importance of Occupational Medicine was the coordination of COVID-19 vaccination campaigns within companies, as well as the identification and monitoring of workers belonging to groups at increased risk of severe disease. Without the data generated through occupational medical examinations, identifying these workers and implementing workplace accommodations would have been considerably more limited.

Section 7.3.2.1 of NR-7 establishes that the PCMSO must include medical evaluations arising from workers’ spontaneous health care requests as well as preventive measures based on clinical findings identified during occupational medical examinations. This provision is consistent with Section 7.5.4.c, which requires the establishment of “criteria for interpreting medical examination findings and planning the corresponding clinical management.”

Likewise, Section 7.5.4.e establishes that the PCMSO must function as a dynamic program, subject to continuous improvement based on the analysis of indicators presented in its analytical report.

An analysis of the practical application of NR-7 demonstrates that the scenario described in Bill No. 1,083/2021, characterized by superficial and unnecessary occupational medical examinations, tends to occur when the strategic potential of the PCMSO is poorly understood or underutilized, when deficiencies exist in program planning, when examinations are performed with inadequate technical quality, and when integrated preventive measures are absent.

This context perpetuates a cycle of misinformation, disinterest, and limited engagement among workers, employers, and health professionals regarding workers’ health and the objectives of occupational medical examinations. As a result, the credibility of Occupational Medicine is diminished, the implementation of evidence-based preventive measures is hindered, and compliance with occupational health legislation becomes less effective.

Within organizations, occupational health systems evolve progressively through consensus-building among multiple stakeholders, including workers, business leaders, the SESMT, the Internal Commission for Accident Prevention, labor unions, and the Labor Prosecution Office. Throughout this process of institutional improvement, the physician coordinating the PCMSO, together with the other members of the SESMT, advances occupational health practices in accordance with technical expertise, ethical commitment, institutional credibility, and organizational leadership support.

An analysis of the current version of NR-7, together with best practices already implemented by numerous organizations, demonstrates that occupational medical examinations are neither an end in themselves nor limited to their medicolegal functions. Rather, they constitute an epidemiological tool integrated into the PCMSO and other initiatives aimed at protecting both individual and occupational health, thereby contributing to the effective implementation of occupational health legislation, the promotion of workers’ health, and the protection of workers’ rights.

### THE SOCIAL AND STRATEGIC RELEVANCE OF OCCUPATIONAL MEDICAL EXAMINATIONS IN THE PRACTICE AND ADVANCEMENT OF OCCUPATIONAL MEDICINE

Occupational medical examinations are conducted in accordance with the ethical principles governing the medical profession and the duty of confidentiality established by the Code of Medical Ethics (CME), enabling the management of sensitive health information in compliance with the Brazilian General Data Protection Law. Data obtained during occupational medical examinations support the preparation of the PCMSO analytical report, enabling the identification of health needs, the planning of individual and occupational health care pathways, and the continuous improvement of workers’ health management.

The PCMSO and occupational medical examinations are not ends in themselves but rather represent one of the primary entry points for implementing actions and health care pathways integrated into the program. In this context, occupational medical examinations fulfill complementary functions at the individual, collective, and organizational levels of health management [^11^].

Understanding the relationship between work and health is a continuously evolving field of knowledge that presents distinct characteristics according to the productive sector, occupational activity, and organizational context. Consequently, Occupational Medicine requires local epidemiological analyses and qualified listening to workers, in accordance with CFM Resolution No. 2,323/2022. Active listening and the longitudinal follow-up of workers’ health promote the establishment of a physician–worker relationship, deepen the understanding of the interactions among health, disease, and work, and guide the planning of actions integrated into the PCMSO.

For example, investigating causality in cases of lateral epicondylitis illustrates how epidemiological analysis can inform decision-making at the individual, collective, and organizational levels. Evaluating the epidemiological profile of this condition makes it possible to determine whether cases are meaningfully associated with occupational activities or whether individual risk factors predominate. These findings strengthen causal assessments in individual cases, guide decisions regarding the need for workplace accommodations, and help establish priorities for both interventions targeting individual risk factors, such as hypertension, diabetes mellitus, and smoking [^8^], and ergonomic measures aimed at improving workplace biomechanical conditions [^9^].

Similarly, integrating epidemiological analyses with AET is essential for identifying patterns of disease and work-related complaints, particularly in cases of mental disorders associated with organizational factors such as high psychological demands, excessive performance pressure, and other psychosocial hazards [^12^]. The quality of these analyses depends directly on the quality of occupational medical examinations and the electronic health record systems used, since more complete and reliable data allow more accurate identification of opportunities to improve work environments and processes, thereby enhancing workers’ health management.

Occupational medical examinations also offer unique preventive potential for both public health and occupational health by providing access to asymptomatic workers who often do not seek or adhere to conventional health care services despite already presenting obesity, diabetes mellitus, dyslipidemia, hypertension, depression, anxiety, unhealthy dietary habits, smoking, harmful alcohol use, or other risk factors for disease. In some organizations, the PCMSO integrates occupational medical examinations with clinical care teams, health promotion, well-being and quality-of-life programs, and programs for the prevention and treatment of tobacco, alcohol, and other psychoactive substance use [^13^].

Within the context of periodic medical examinations, some PCMSOs incorporate clinical care activities, including clinical diagnosis and therapeutic prescribing by occupational physicians, thereby promoting primary and secondary prevention while expanding access to the early diagnosis and treatment of diseases that are common among working populations. Regardless of disease etiology, workers with health conditions tend to experience reduced productivity, greater difficulty entering and remaining in the workforce, and higher rates of absenteeism, with negative consequences for their well-being, their families, and the sustainability of health care and social security systems.

**Figure 1.**
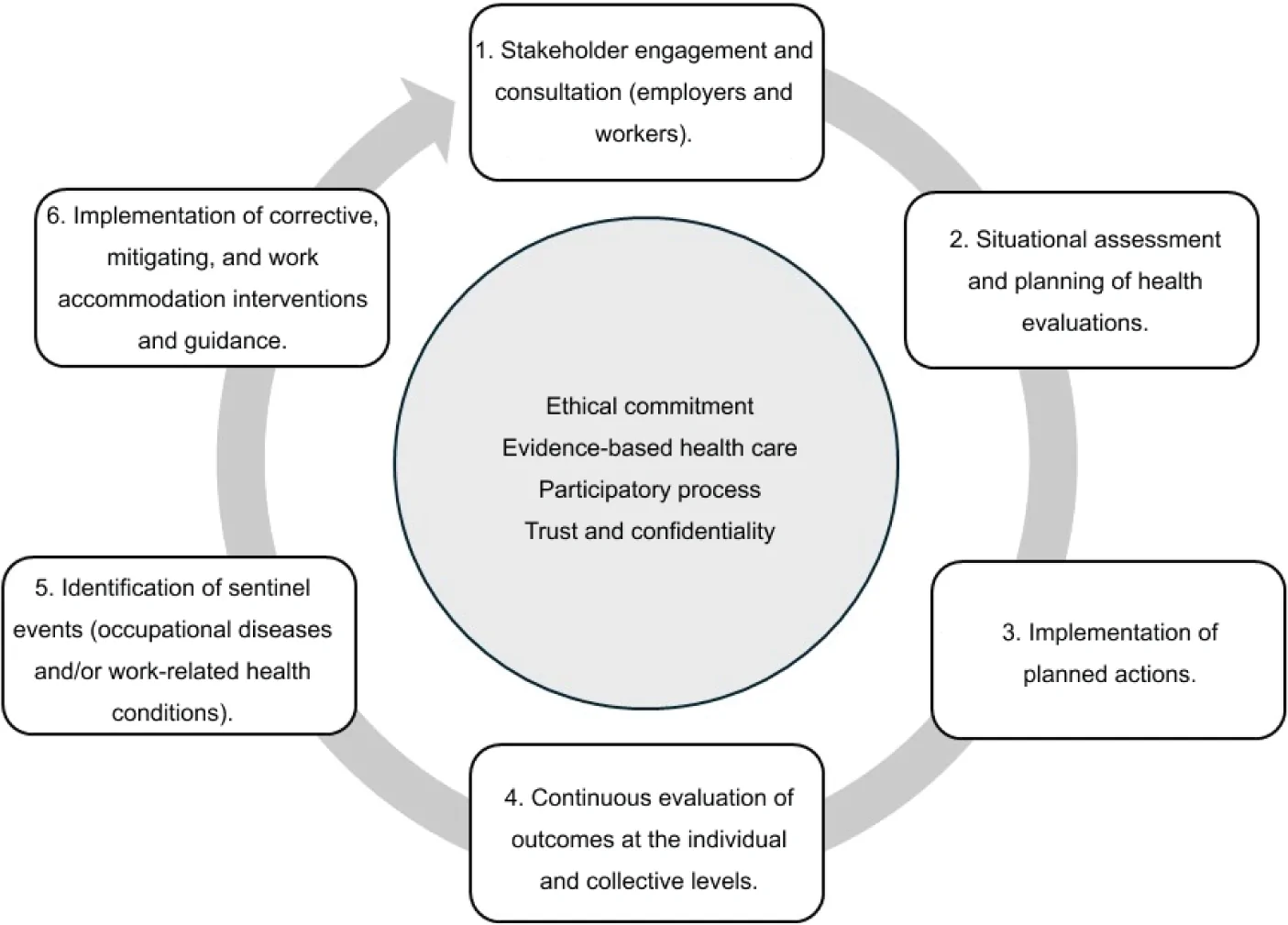
Virtuous cycle of continuous improvement driven by the Brazilian Occupational Health Medical Control Program and occupational medical examinations.

Source: Prepared by the authors.

In this context, occupational medical examinations contribute to the development of epidemiological consensus that can guide priority setting, the planning of individual and occupational health interventions, and the more efficient allocation of resources for workers’ health management, thereby establishing a virtuous cycle that strengthens occupational health systems. Figure 1 illustrates this virtuous cycle, based on the principles established by the NRs.

Conversely, the lack of epidemiological data undermines the development of consensus regarding workers’ health status, making strategic planning, intervention prioritization, and the rational allocation of resources more difficult. As a result, the lack of systematic structures for monitoring and mitigating work-related health conditions becomes more common, while the technical robustness of the Brazilian Risk Management Program (PGR, in Portuguese), the PCMSO, analytical reports, and occupational medical examinations themselves is weakened. This scenario may negatively affect organizational performance indicators, including by increasing vulnerability to labor litigation resulting from both the absence of documented preventive measures and the reduced technical robustness of occupational health documentation.

### RECOMMENDATIONS FOR PROMOTING BEST PRACTICES IN OCCUPATIONAL MEDICAL EXAMINATIONS

Recognizing that the PCMSO and occupational medical examinations are not ends in themselves but rather an entry point for integrated actions aimed at health promotion, disease prevention, health surveillance, and individual and occupational health care is the first step toward understanding the consequences of poorly designed or inadequately implemented programs, whose adverse effects extend to employers, workers, occupational physicians, and public health itself.

In this context, organizations such as the National Association of Occupational Medicine, the Brazilian Association of Occupational Medicine, the Brazilian Association of Occupational Safety and Health Companies, the Occupational Safety and Health Management Association, the CFM and Regional Councils of Medicine, among others, can play a strategic role in developing, consolidating, and disseminating technical consensus statements and protocols establishing quality standards for the PGR, the PCMSO, and occupational medical examinations. Likewise, these organizations can contribute to the development and dissemination of guidelines on ergonomics, AET, primary and secondary prevention in workers’ health, and the investigation and mitigation of work-related diseases, taking into account the specific characteristics of each occupational activity.

CFM Resolutions No. 2,323/2022, No. 2,430/2025, No. 2,056/2013, No. 2,153/2016, and No. 2,217/2018 constitute important references for evaluating the ethical and technical compliance of the PCMSO. In particular, CFM Resolution No. 2,153/2016 provides updated inspection protocols for health care services, including Occupational Medicine services, establishing objective criteria for evaluating their structure and operation.

A comprehensive analysis of the current legislation, NR-7, CFM resolutions, and successful experiences already implemented in Occupational Medicine demonstrates that inadequately structured PCMSOs and occupational medical examinations promote noncompliance with the legislation, the NRs, CFM resolutions, and the CME. Furthermore, they reinforce the mistaken perception that the primary purpose of occupational medical examinations is merely the issuance of the Occupational Health Certificate, thereby undermining the strategic role of the SESMT and the PCMSO as instruments for generating epidemiological knowledge and planning actions aimed at promoting workers’ health and preventing work-related diseases.

Consistent with the principle of comprehensive care established in the National Policy on Workers’ Health by Ordinance No. 1,823 of August 23, 2012, the PCMSO and occupational medical examinations should adopt a worker-centered approach focused on comprehensive health, as has already been observed in some organizations. In this context, occupational medical examinations make it possible to identify workers with hypertension, diabetes mellitus, obesity, smoking, depression, anxiety, exposure to violence, and work-related diseases who often do not seek medical care on their own and who are at increased risk of impaired work capacity, absenteeism, and greater use of health care and Social Security services. Thus, the objectives and potential of occupational medical examinations extend beyond their medicolegal functions related to assessing fitness for work and establishing causality between disease and work.

Occupational medical examinations also represent an important source of primary data for occupational health research. The generation and publication of scientific studies based on these data can strengthen the technical and scientific credibility of Occupational Medicine, expand knowledge regarding the relationships between work and disease, support the development of updated technical consensus statements, guide the planning and evaluation of the effectiveness of programs aimed at preventing and mitigating work-related health conditions, and stimulate observational research, including case reports, case-control studies, and cohort studies, thereby advancing scientific knowledge and strengthening workers’ health policies for the benefit of Brazilian society.

## CONCLUSIONS

The authors advocate the rejection of Bill No. 1,083/2021, arguing that its approval would represent a setback in the historical evolution of occupational medical examinations by weakening the technical and epidemiological role of Occupational Medicine and the PCMSO in promoting actions to prevent and mitigate work-related health conditions. Furthermore, the proposal would compromise compliance with the legislation and workers’ access to their rights by disregarding the occurrence of work-related diseases among young workers and those engaged in administrative occupations, while also overlooking the strategic importance of a preventive approach focused on workers’ comprehensive health for public health. The authors also emphasize the need to promote the development and dissemination of technical consensus statements, protocols, guidelines, and scientific publications to support improvements in the design of the PCMSO and occupational medical examinations, as well as the implementation of individual and occupational health care pathways based on the needs identified during occupational medical examinations.

## Data Availability

The data supporting the findings of this study are available within the article.
